# Targeting miRNA and using miRNA as potential therapeutic options to bypass resistance in pancreatic ductal adenocarcinoma

**DOI:** 10.1007/s10555-023-10127-w

**Published:** 2023-07-25

**Authors:** Mahrou Vahabi, Bilal Dehni, Inés Antomás, Elisa Giovannetti, Godefridus J. Peters

**Affiliations:** 1 Department of Medical Oncology, Amsterdam UMC, location VUMC, Vrije Universiteit Amsterdam, Cancer Center Amsterdam, Amsterdam, Netherlands; 2 Cancer Pharmacology Lab, Fondazione Pisana per La Scienza, Pisa, Italy; 3 Department of Biochemistry, Medical University of Gdansk, Gdansk, Poland

**Keywords:** PDAC, oncomiRs, TsmiRs, Chemoresistance, miRNA-based therapies

## Abstract

Pancreatic ductal adenocarcinoma (PDAC) is a highly aggressive disease with poor prognosis due to early metastasis, low diagnostic rates at early stages, and resistance to current therapeutic regimens. Despite numerous studies and clinical trials, the mortality rate for PDAC has shown limited improvement. Therefore, there is a pressing need to attain. a more comprehensive molecular characterization to identify biomarkers enabling early detection and evaluation of treatment response. MicroRNA (miRNAs) are critical regulators of gene expression on the post-transcriptional level, and seem particularly interesting as biomarkers due to their relative stability, and the ability to detect them in fixed tissue specimens and biofluids. Deregulation of miRNAs is common and affects several hallmarks of cancer and contribute to the oncogenesis and metastasis of PDAC. Unique combinations of upregulated oncogenic miRNAs (oncomiRs) and downregulated tumor suppressor miRNAs (TsmiRs), promote metastasis, characterize the tumor and interfere with chemosensitivity of PDAC cells. Here, we review several oncomiRs and TsmiRs involved in chemoresistance to gemcitabine and FOLFIRINOX in PDAC and highlighted successful/effective miRNA-based therapy approaches in vivo. Integrating miRNAs in PDAC treatment represents a promising therapeutic avenue that can be used as guidance for personalized medicine for PDAC patients.

## Introduction

Pancreatic ductal adenocarcinoma (PDAC) is a virulent cancer type and the third leading cause of cancer-related death in the United States with a dismal 5-year overall survival (OS) of 11% [1, 2]. A number of reasons account for this poor prognosis including early metastasis, high local recurrence rate and chemoresistance [3]. The absence of specific symptoms in the initial phases of the disease, combined with the lack of effective screening methods, contribute to the identification of advanced-stage cases in approximately 80% of patients, with infiltration to proximal lymph nodes and vessels as well as metastatic spread to the liver/peritoneum [2]. Surgical resection of the pancreas is the only potentially curative modality for PDAC, but detection at later stages limits the number of patients benefiting from resection at the time of diagnosis. Nonetheless, a small subgroup of patients are alive and cancer-free after 5 years from surgery [4].

A few therapeutic options are available for patients with metastatic PDAC. Gemcitabine as monotherapy, or in combinations (mostly with nab-paclitaxel), shows some clinical benefit in such advanced settings but the disease control remains limited, with < 15% of patients progression-free at 6 months from diagnosis [3, 4]. Several attempts have been made to increase the effectiveness of treatment by combining gemcitabine with other agents, such as the oral prodrug formulation of 5-FU, S-1, or the EGFR-targeted drug erlotinib, but these regimens yielded only marginal improvements. Conversely, the FOLFIRINOX regimen, consisting of 5-FU, leucovorin, irinotecan, and oxaliplatin, resulted in a substantial increase in OS compared to gemcitabine monotherapy, with a median OS of 11.1 months, but increased toxicity [5, 6].

Genetic investigations of PDAC have unveiled shared molecular characteristics, such as the presence of KRAS gene mutations in over 90% of patients, and inactivation or deletion mutations in tumor suppressor genes like TP53, CDKN2A, and SMAD4 [6]. In addition, accumulating evidence suggests that PDAC is characterized by aberrations of genes that function through key pathways, leading to the formation of complex signaling networks [7]. Jones et al. [8] were the first to define 12 “core” signaling pathways contributing to tumorigenesis and disease progression, which could serve as potential targets. The best hope for the development of more effective anticancer agents is either by targeting one of these altered signaling pathways and/or deciphering mechanisms regulating their gene expression [9].

**Fig. 1:**
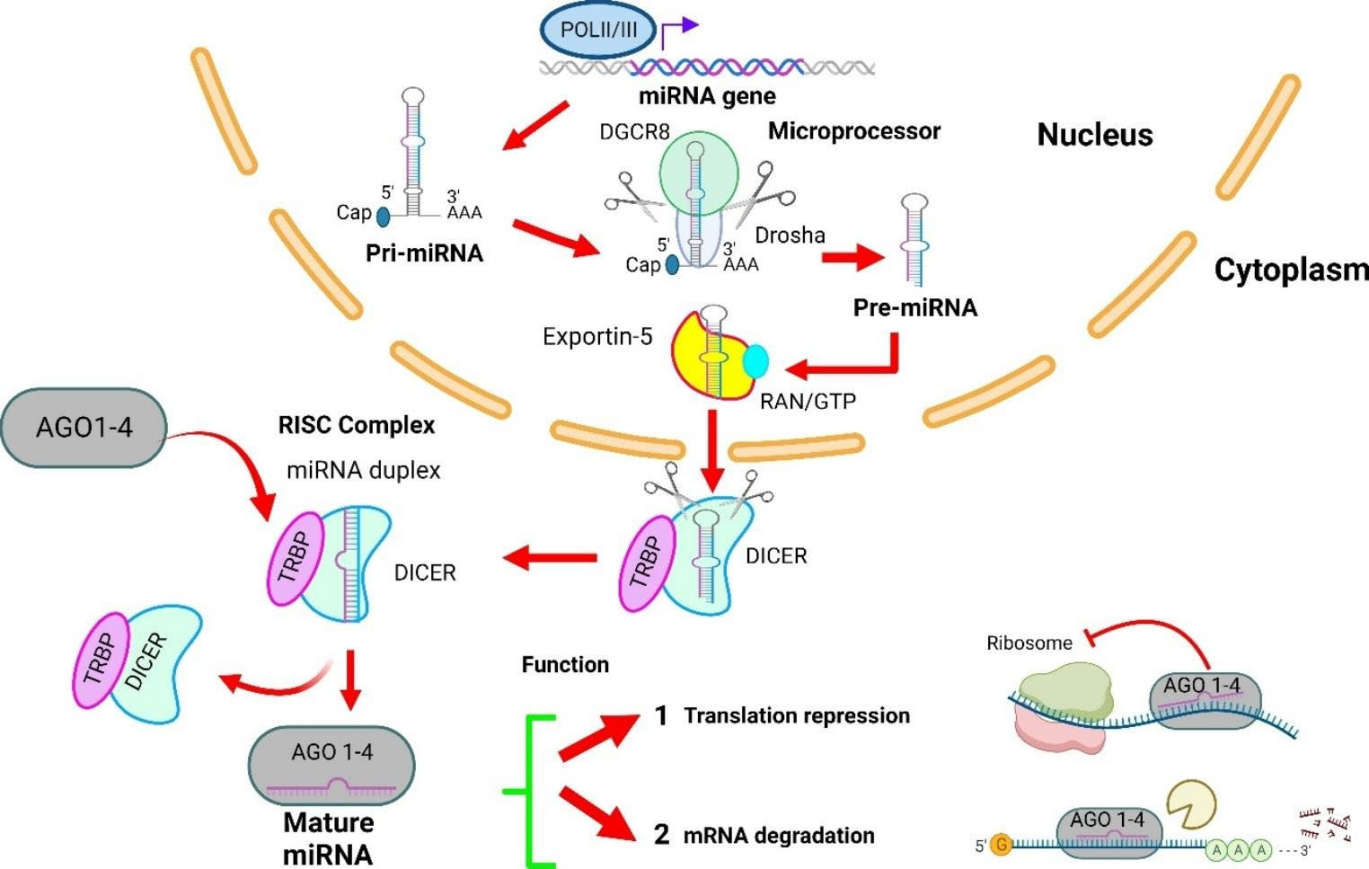
miRNAs biogenesis and mechanisms of gene silencing in a nutshell. miRNAs are transcribed from DNA by RNA polymerase II /III to form single-stranded looped primary transcript (pri-miRNA). These pri-miRNAs are cleaved by Drosha and DGCR8 to release the ~ 70-nucleotides hairpin RNA called pre-miRNA which is transported to the cytoplasm via exportin-5. Next, a miRNA duplex of 22 nucleotides is generated by Dicer. This duplex is unwound into mature single-stranded mature miRNA that incorporates in the RNA-induced silencing complex (RISC) where it forms complementary pair with a certain mRNA. Depending on the degree of complementarity, the mRNA is degraded or translationally repressed. miRNAs can also be translocated to dendrites and axons

Remarkably, the relatively recent discovery of microRNAs (miRNAs) has provided novel insights potentially elucidating the existing gap between genotype and phenotype. miRNAs are a class of small non-coding regulatory RNAs (ncRNAs) with sizes of 17–25 nucleotides with an important role in the post-transcriptional repression of messenger RNA (mRNA) in diverse eukaryotic lineages [10, 11]. More than one third of all genes are regulated by miRNAs which demonstrates their relevance in diverse physiological and developmental processes, such as angiogenesis [12]. Biogenesis of miRNAs starts with transcription from DNA as pri-miRNAs by RNA polymerase II and RNA polymerase III (Fig. 1). Pri-miRNAs contain a single or multiple (miRNA cluster) stem-loop structures and upon synthesis, RNase III (Drosha) and the DGCR8 (DiGeorge critical region 8) will process the pri-miRNAs into a single stem-loop containing 60–70 nt RNAs [11, 12]. These pre-miRNAs are transported to the cytoplasm via a nuclear export factor Exportin-5/Ran-GTP. In the cytoplasm, several proteins such as RNase III endonuclease, Dicer/TRBP and Ago2 allow a series of cuts that generate a mature miRNA duplex. Consequently, the duplex is unwounded by a helicase into a mature single-stranded miRNA that incorporates in the RNA-induced silencing complex (RISC). This complex is directed to target mRNA by binding to the 3’-untranslated region (3’-UTR) of target mRNA. As a result, mRNA translation is inhibited or mRNA is targeted for degradation.

MiRNA dysregulation is a common occurrence in various cancers, including PDAC [13], This dysregulation can arise from multiple mechanisms, including (1) miRNA localization in cancer-associated genomic regions, (2) epigenetic modulation of miRNA genes, and (3) disruptions in miRNA processing genes and proteins[14–16]. Moreover, genetic aberrations and transcriptional alterations also contribute to the deregulation of miRNAs in PDAC and other cancers.

At the cellular level, certain miRNAs primarily exert their influence on cancer cell-intrinsic processes and pathways, while others predominantly act within specific cellular compartments of the tumor microenvironment (TME) to regulate the functions of cancer-associated fibroblasts (CAFs) and immune cells. Moreover, these miRNAs can also modulate the functions of other cell types through intercellular communication mediated by the transfer of extracellular vesicles (EVs) [17]. For example, a study by Han et al. conducted in vitro (on PDAC cells SW1990 and PANC-1) and in vivo (PANC-1 tumor xenografts in BALB/c nude mice) demonstrated that miR-331-3p was enriched in EVs derived from CAFs and could be transferred to PDAC cells SW1990 and PANC-1. Within the PDAC cells, miR-331-3p suppressed the axis involving the scavenger receptor class A member 5 (SCARA5) and focal adhesion kinase, thereby promoting tumor cell proliferation, migration, and invasion. Consistent with these findings, miR-331-3p was found to be upregulated in PDAC tissues compared to normal tissues and was associated with lower survival rates. Additionally, SCARA5 expression was decreased in cancer tissues and correlated with a poor prognosis [18].

At the molecular level, miRNA-mediated regulation frequently converges on shared targets and functions, leading to overlapping effects. This convergence is particularly evident in PDAC core signaling pathways such as TGF-β, JAK/STAT, PI3K/AKT, and NF-κB, as summarized in previous reviews [19, 20]. MicroRNAs can collectively regulate these pathways by targeting multiple components within them, modulating the expression of key signaling molecules, and influencing downstream signaling events. This interconnectedness and cross-talk among miRNAs and core signaling pathways highlight the intricate regulatory network orchestrated by miRNAs in cellular processes as well as in disease pathogenesis. Therefore, understanding these overlapping functions and targets provides valuable insights into the complex mechanisms underlying miRNA-mediated regulation and opens up possibilities for the development of novel therapeutic approaches targeting these interconnected pathways.

The differential expression of miRNA in malignancies has led to their evaluation as biomarkers for diagnostic, prognostics and therapeutic importance, using both tissue and liquid biopsies [21]. Notably, miRNAs have been shown to be more stable inside membrane-bound vesicles in the extracellular environment called exosomes. Mounting evidence has revealed that miRNAs in EVs are intensely connected with various cancers, including PDAC [22]. Varieties of miRNAs are indeed released into the body fluids via EVs depending on the normal physiological or pathological conditions of the body [23]. To harness the potential of miRNAs for enhancing PDAC management, understanding the molecular basis of their pathological pro-tumoral effects is crucial. In this regard, miRNAs can be categorized into two main groups: oncogenic miRNAs (oncomiRs) that suppress the expression of tumor suppressor genes, thereby promoting PDAC oncogenesis, and tumor suppressor miRNAs (TsmiRs) that inhibit the expression of oncogenes involved in PDAC development. This classification provides valuable insights into the functional roles of miRNAs in PDAC and lays the foundation for exploring their clinical implications [24, 25]. Unique combinations of upregulated oncomiRs and downregulated TsmiRs characterize the tumor, its metastatic capacity and interfere with the expression of multiple target mRNAs causing variation in chemosensitivity of PDAC cells. In particular, these miRNAs alter cellular response to anticancer drugs via modulation of drug efflux and influx, cell cycle, survival pathways, and/or apoptotic response. In this review, we provide a summary of relevant oncomiRs (Table 1) as well as TsmiRs that are mechanistically involved to the currently used chemotherapeutic agents i.e., gemcitabine/nab-paclitaxel and FOLFIRINOX [24]. Because of the number of preclinical studies as well as studies including clinical specimens [26, 27], a separate table summarizes the studies on miR-21 (Table 2).

We discuss in detail the therapeutic potential of miRNAs, highlighting novel miRNA delivery approaches that can be applied to use the increasing knowledge on miRNAs to potentially improve treatment of PDAC. In particular, the presentation of miRNAs in the following paragraphs is based on their categorization in oncomiRs and tsmiRs, and on the number of studies reported in the context of PDAC.

**Table 1:** Overview of oncogenic and tumor suppressor miRNAs in PDAC

miRNA	Role	Target(s)	Study	Downstream effect(s)	Main preclinical model(s)	Ref.
miR-21	OncomiR	PDCD4, TIMP3, PTEN	In clinical samples, in vitro and in vivo	Enhanced proliferation, progression of cell cycle, metastasis and gemcitabine resistance	Human PDAC cell lines, tissues and PDAC xenograft mouse models. Additional studies are detailed in the Table 2	[26, 27]
miR-155	OncomiR	TP53INP1, SOCS1	In clinical samples, in vitro and in vivo	Enhanced tumor growth, invasion and migration and exosome-mediated resistance	Human PDAC cell lines (PANC-1, PATU8988, MIA PaCa-2, PSN1); PANC-1 subcutaneous xenograft model in NOD/SCID mice	[28, 29]
miR-10a-5p	OncomiR	TFAP2C	In clinical samples, in vitro and in vivo	Enhanced migration, invasion and metastasis	Human PDAC cell lines (AsPC-1, BxPC-3, MIA PaCa-2, PANC-1, Su86); PDAC subcutaneous xenograft model in BALB/c mice	[30]
miR-10b	OncomiR	TIP30	In clinical samples, in vitro and in vivo	Increased EGF levels, invasiveness and metastasis	Human PDAC cell lines (COLO-357, PANC-1, AsPC-1, T3M4; MIA PaCa-2, BxPc-3); T3M4 orthotopic model in athymic mice	[31–33]
miR-342-3p	OncomiR	KLF6	In clinical samples, in vitro and in vivo	Pro-survival and gemcitabine resistance	Human PDAC cell lines (PANC-1, MiaPaCa-2) and pancreatic ductal immortalized cells (HPDE6c7); xenograft model in athymic mice	[34]
miR-296-5p	OncomiR	BOK	In clinical samples, in vitro	Decreased apoptosis, enhanced metastasis and chemoresistance to gemcitabine and 5FU	Human PDAC cell lines (PANC-1, MIA PaCa-2, PK-1, PK-8, PK-45 H)	[35]
miR-17-5p	OncomiR	RBL2, Bim, beclin-1	In vitro and in vivo	Decreased apoptosis and chemoresistance to gemcitabine and paclitaxel	Human PDAC cell lines (AsPC-1, BxPC-3, HPDE6c7, MIA PaCa-2, PANC-1, CFPAC-1) and their xenograft models in athymic mice	[36–39]
miR-181b-5p	OncomiR	ATM	In vitro	Modulation of DDR and resistance to FOLFIRINOX	Human PDAC cell lines (PANC-1, SUIT2) and primary cultures (PDAC3)	[40]
miR-211	Ts-miR	RRM2	In clinical samples, in vitro	Increased antiproliferative effects of gemcitabine	Human PDAC cell lines, primary cultures and subclones of human PDAC SUIT2 cells (SUIT2-007, SUIT2-028)	[41, 42]
miR-34a	Ts-miR	Bcl2, Notch 1/2	In clinical samples, In vitro and in vivo	Inhibition of PDAC stem cell renewal	Human PDAC cells (MiaPaCa-2 and BxPC3); MIA PaCa-2 subcutaneous xenografts in athymic NCr-nu/nu nude mice	[43–45]
miR-146a-5p	Ts-miR	TRAF6	In vitro and in vivo	Suppression of PDAC cell proliferation and increased sensitivity to gemcitabine	Human PDAC cells (Capan-1, MiaPaCa-2, BxPC-3, SW199, PANC-1) and xenografts in BALB/c mice	[46]
miR-30a-5p	Ts-miR	FOXD1	In vitro and in vivo	Increased sensitivity to gemcitabine	Human PDAC cell lines (Panc-1, BxPC-3, MiaPaCa-2), and HPDE6c7; BxPC-3 subcutaneous xenograft in athymic mice	[47]
miR-125a-5p	Ts-miR	Fyn	In vitro	Increased antitumor effects and suppression of EMT	Human PDAC cell lines (PATU8988 T, PANC-1)	[48]
miR-216b	Ts-miR	ROCK1	In vitro	Inhibition of proliferation, migration and invasion	Human PDAC cell lines (PANC-1, Bxpc-3, Sw1990, Aspc-1) and HPDE6c7	[49]

**Abbreviations**: 5FU, 5-fluorouracil; ATM, ataxia telangiectasia mutated; bcl-2, B-cell lymphoma 2; Bim, bcl-2-like protein 11; BOK, bcl-2 related ovarian killer; FOXD1, forkhead box d1; KLF6, Krüppel-like factor 6; Notch1/2, neurogenic locus notch homolog protein ½; PDCD4, programmed cell death protein 4; PTEN, phosphatase and tensin homolog; RBL2, retinoblastoma-like protein 2; ROCK1, rho-associated coiled-coil-containing protein kinase 1; RRM2, ribonucleoside-diphosphate reductase subunit M2; SOCS1, suppressor of cytokine signaling 1; TFAP2C, transcription factor AP-2 gamma; TIMP3, metalloproteinase inhibitor 3; TIP30, Tat-interacting protein 30; TP53INP1, tumor protein p53 inducible nuclear protein 1; TRAF6, TNF receptor-associated factor 6

### OncomiRs in PDAC

OncomiRs are the group of microRNAs involved in the regulation of numerous genes that contribute to proliferation, differentiation, migration and invasion. Many oncomiRs have been associated with oncogenesis, metastatic and invasive capabilities of PDAC as well as resistance to current treatments. The following section discusses nine of them (Table 1).

#### miR-21

MicroRNA-21 was reported to be overexpressed and contributing to invasion, metastasis and gemcitabine resistance in PDAC [26, 50–55]. Giovannetti and colleagues [50] evaluated miR-21 expression in cells in primary PDAC cell cultures, fibroblasts and a normal pancreatic ductal cell line as well as tumor tissues from 81 PDAC patients. Tumor tissue was isolated by laser microdissection (to minimize stromal contamination). Patients treated with gemcitabine in a metastatic or adjuvant setting and with high miR-21 expression had a significantly shorter OS compared to patients with a low miR-21. Hwang et al. [51] found similar data in a Korean and Italian cohort, but also observed that a high miR-21 correlated with the poor outcome associated with 5FU-based adjuvant therapy. A common criticism about the reported studies on miR-21 in PDAC is that the sample sizes are relatively small, which may limit the generalizability of the findings. This could lead to biased or incomplete results, as the sample size may not be representative of the larger PDAC patient population. However, a more recent study included 686 tissue samples to evaluate whether miR-21 is a predictor of survival. This is the largest population ever investigated for the analysis of a miRNAs as a potential biomarker in PDAC specimens. The tissue expression of miR-21 was evaluated by chromogenic in situ hybridization (CISH). By applying this method to tissue microarrays of well-annotated PDAC cohorts of patients, the researchers showed that the epithelial expression of miR-21 is an independent robust prognostic biomarker in PDAC, prompting prospective trials for further validation and application in the clinical setting [56]. The above-mentioned studies also showed that PDAC cells treated with gemcitabine and transfected with pre-miR-21 became less sensitive and had a decreased apoptosis induction, while transfection with anti-miR-21 enhanced the gemcitabine sensitivity. This could be explained by miR-21-mediated downregulation of the tumor suppressor PTEN, leading to the activation of PI3K/Akt/mTOR pathway [50]. Activation of the Akt pathway is a common survival mechanism of cancer cells after being exposed to DNA-targeted therapy or radiation [57], rendering the PDAC cells resistant to gemcitabine. Wei et al. [27] observed that a reduced miR-21 expression (leading to increased PTEN and decreased PDC4) would also increase the sensitivity to 5FU. This suggests that miR-21 confers resistance to both gemcitabine and 5FU, and the in vitro findings might explain the data in the PDAC patients.

More recently, in 177 patients with advanced pancreatic cancer, treated with gemcitabine, high serum levels of miR-21 were significantly correlated with a shorter time-to-progression (TTP) and lower OS. In addition, Nagao et al. [26] demonstrated that miR-21 downregulated its molecular targets programmed cell death 4 (PDCD4) and tissue inhibitor of metalloproteinase (TIMP3) in PDAC, potentially explaining the tumor-invasive behavior of PDAC and poor survival of patients with high miR-21 levels Altogether, these data suggest that high miR-21 expression is related to poor outcome of both gemcitabine and 5FU-based therapy.

Strikingly, multiple studies showed that miR-21 targets important tumor suppressor genes as well as genes involved in carcinogenesis, such as PTEN, PDCD4, and RECK. Therefore, miR-21 is a potential molecular biomarker for diagnosis, prediction, and prognosis, as well as a new therapeutic target [58]. Thus, further insights into the mechanisms obtained from preclinical models are highly valuable for advancing and refining potential clinical applications, as described in Table 2.

Schipper et al. conducted a study investigating the global loss of miR-21 in genetically engineered mouse models (GEMM) of PDAC driven by K-Ras and lacking p53. Intriguingly, the loss of miR-21 enhanced tumor initiation and progression, leading to aggressive locally advanced invasive carcinoma and early mortality. These findings support the tumor-suppressive role of miR-21 in biologically-relevant in vivo models of pancreatic tumorigenesis and imply potential therapeutic applications of miR-21 inhibitors. Remarkably, loss of miR-21 enhanced tumor initiation via mucinous cystic neoplastic lesions and progression to locally advanced invasive carcinoma from which animals precipitously succumbed at an early age. If miR-21 activity has a similar effect in human PDAC as observed in these studies on GEMM models, this could have clinical implications for therapeutic use of miR-21 inhibitors as chemopreventive agents [59].

Most recently, miR-21 also emerged as an important regulator in the activation of CAFs. Zhang et al. evaluated the relationship between CAF activation, miR-21 expression, and drug resistance using tumor samples from PDAC patients. They also examined the roles of miR-21 in CAFs in the development of PDAC using an animal model with the Panc02 cell line, murine CAFs, and C57BL/6 mice. Their results indicated that gemcitabine-resistant PDAC patients exhibited higher miR-21 expression and increased CAF activation. The preclinical experiments demonstrated that miR-21 overexpression contributed to CAF activation through the regulation of the PDCD4 gene and to resistance to gemcitabine. Thus, miR-21 was implicated in CAF activation and the development of drug resistance in PDAC [60]. Of note, another recent study Chen evaluated if the metabolic alteration of CAFs occurs via miR-21 remodeling and the effect of this alteration on PDAC cells. Compared to normal fibroblasts, CAFs showed enhanced glucose uptake capacity, lactic acid production, and elevated LDHA, PKM2, and miR-21 expression. Additionally, miR-21 was involved in metabolic alteration of CAFs and affected the development of cancer cells [61].

Finally, an assessment of miR-21 expression in the PDAC cohort from The Cancer Genome Atlas revealed a link between the content of tumor epithelial cells and miR-21 expression in human tumors. This finding supports the need for conducting further studies on miRNA in human specimens, suggesting that miR-21 could be beneficial for detecting early pancreatic intraepithelial neoplasia (PanIN) and intervening in the progression of premalignant pancreatic lesions and other premalignancies driven by KRAS mutations. Indeed, almost all PDAC are initiated by the expression of the driver mutation KRAS. However, KRAS is mutated in in over 90% of human PanIN lesions. Most importantly, studies in KPC mice programmed to recapitulate human PDAC tumorigenesis showed that inhibition of miR-21 intercepted premalignant progression, reverting protumorigenic functionalities to baseline levels and improved survival in already established PDA. Importantly, early systemic miR-21 inhibition completely. Thus, miR-21 may be useful for early PanIN detection and for preventing developing premalignant pancreatic lesions as well as other KRAS-driven premalignancies [62].

**Table 2:** Preclinical and clinical studies on the role of miR-21 in PDAC

Target(s)	Type of study	Main preclinical model(s)	Downstream effect(s)	Ref.
PTEN	In vitro and in clinical samples	Human PDAC cell lines and primary cultures	Gemcitabine chemoresistance and inhibition of apoptosis	[50]
PDCD4, TIMP3	In vitro and in clinical samples	Human pancreatic and PDAC tissue samples	Tumor progression	[26]
FasL, PTEN, PDCD4	In vitro and in vivo	Human PDAC cell lines (PANC-1, BxPC3), and xenografts in BALB/c-nu/nu nude mice	Chemoresistance, enhanced resistance to gemcitabine-induced apoptosis	[55]
PTEN, PDCD4	In vitro	Human PDAC cell lines (PATU8988, PANC-1)	5-FU chemoresistance, migration and invasion	[27]
PDCD4, PTEN, Sprouty-1/-2	In vitro and in vivo	Genetically engineered mouse models of PDAC	Promotes cell growth, required for TGF-β signaling, carcinogenesis, tissue fibrosis and inflammation	[59]
PTEN, PDCD4, RECK, STAT3	In vitro, in vivo, and in clinical samples	Different tumor types: breast, prostate, hepatocarcinoma, colon cancer and PDAC cell lines and mouse models	Enhanced cell proliferation, migration and invasion, interference in apoptosis	[58]
PDCD4	In vitro and in vivo	PDAC cells MIA Paca-2 cells, Panc02 and their xenograft mouse model, PDAC tissue samples	Promotes activation of CAFs, chemoresistance, cell invasion	[60]
PTEN	In vitro	Human PDAC cells (BxPc-3 and PANC-1)	Promotes cell invasion, metabolic alteration of CAFs, development of cancer cells	[61]
PTEN, SDCD4, Sprouty2, TPM1, RASA1	In vivo	Genetically engineered mouse	Promotes EMT, invasion and migration	[62]
-	In vitro and bioinformatics analysis	Human PDAC cells (BxPc3, HPAF-II, HPAC, PANC-1, PL45)	Enhanced cell proliferation and gemcitabine resistance	[51]
-	In clinical samples	Bioinformatics analysis in PDAC tissue samples	-	[52]
-	In clinical samples, in vitro/vivo	HPDE, BxPC-3, MIA PaCa-2 cells, and orthotopic BxPC-3 in NOD/SCID IL2Rγ null mice	Poor prognosis, metastasis and invasion	[53]
-	*In clinical samples*	Pancreatic and PDAC tissue samples	Poor clinical outcome, and chemoresistance	[54]

**Abbreviations**: 5FU, 5-fluorouracil; CAF, cancer associated fibroblasts; EMT, epithelial-to-mesenchymal transition; PDCD4, programmed cell death protein 4; PTEN, phosphatase and tensin homolog; RASA1, RAS p21 protein activator 1; RECK, Reversion-inducing cysteine-rich protein with Kazal motifs; STAT3, signal transducer and activator of transcription 3; TIMP3, metalloproteinase inhibitor 3; TPM1, tissue Inhibitor of metalloproteinases 3

#### miR-155

Several studies highlighted the significant role of miR-155 in the aggressive nature of PDAC and its resistance to gemcitabine, as previously reviewed [63]. A representative study was conducted by Greither et al. [28] on 56 microdissected PDAC cases where they measured the levels of miR-155, miR-203, miR-210, miR-216, miR-217, and miR-222 by quantitative RT-PCR. Their findings revealed that higher expression of miR-155, miR-203, miR-210, and miR-222 was associated with a 6.2-fold increased risk of death related to tumors compared to individuals with lower expression levels. Notably, Mikamori et al. [29] demonstrated that prolonged exposure to gemcitabine resulted in increased expression of miR-155 in PDAC gemcitabine-resistant PANC-1 cells. Furthermore, they observed that miR-155-induced EVs secretions and these EVs were taken up by PDAC cells, leading to cellular resistance. This resistance was then transmitted to other PDAC cells, contributing to resistance in those cells as well. These studies demonstrate that miR-155 plays an important role in the aggressive behavior of PDAC and resistance to gemcitabine.

#### miR-10a-5p and miR-10b

miR-10a-5p and miR-10b are microRNAs belonging to the miR-10 family. They are involved in various biological processes and have been implicated in cancer. Xiong et al. [30] observed an increased miR-10-5p expression in gemcitabine-resistant cell lines which promoted PDAC cell migration and invasion. In vivo studies further demonstrated that miR-10a-5p induced resistance to gemcitabine. In situ hybridisation (ISH) showed an upregulation of miR-10a-5p in PDAC tissue samples compared with matched tumor-adjacent tissues. Univariate and multivariate analyses indicated that high miR-10a-5p expression is an independent adverse prognostic factor in PDAC. A low miR-10-5p expression may increase its target, i.e. transcription factor activating protein 2 (TFAP2), leading to sensitization of PDAC cells to gemcitabine, but upregulated p21 levels would be silenced. In addition, a high TFAP2C decreased PDAC cell migration and invasion capability. Survival analysis showed that low TFAP2C expression was also an independent adverse prognostic marker for patients with PDAC. Collectively, miR-10a-5p was associated with poor prognosis and enhanced metastatic capabilities of PDAC cells. Similarly, several studies showed that miR-10b is upregulated in PDAC and correlated with reduced therapeutic response to multiple neoadjuvant therapies, short relapse-time, lower OS and enhanced invasiveness [13, 31–33]. Ouyang et al. [32] demonstrated that miR-10b decreased tat-interacting protein 30 (TIP30) using gene profiling. High levels of miR-10b leading to decreased TIP30, enhanced epidermal growth factor (EGF) stimulating epidermal growth factor receptor (EGFR) phosphorylation and invasiveness of PDAC. Inhibition of EGFR by erlotinib or dual inhibition of the downstream targets PI3K and MEK, blocked the action of miR-10b and EGF. Moreover, in an orthotopic model of T3M4 in athymic mice, the overexpression of miR-10b accelerated both invasiveness and proliferation, ultimately promoting the metastasis of PDAC.

In contrast, Xu et al. reported that miR-10b expression was downregulated in PDAC cells and tissues. They identified E2F7 as a target mRNA of miR-10b, and consequently, the expression of E2F7 was upregulated. Through this mechanism, miR-10b was shown to inhibit the invasion and migration of AsPC1 PDAC cells by regulating the expression of E2F7 [33]. However, it is important to note that these conflicting findings may be attributed to variations in preclinical models and the absence of validation in in vivo models in the latter study.

In a more recent study, Kim et al. showed that the expression of five miRNAs, including miR-10b, was significantly elevated in EVs derived from pancreatic ductal adenocarcinoma. The he combination of this miRNA signature and serum carbohydrate antigen 19 − 9 (CA19-9) effectively differentiated PDAC patients from normal controls [64]. In line with these findings, a meta-analysis study conducted by Jia et al. on the diagnostic performance of EVs biomarkers for PDAC found that miR-10b was frequently reported as an EV-RNA associated with PDAC [65]. Furthermore, Zhao et al. conducted a meta-analysis study revealing that PDAC patients with high expression of various miRNAs, including miR-10b in tissues, exhibited significantly shorter OS [66].

#### miR-342-3p

miR-342-3p, an obesity-associated miRNA, was significantly upregulated in gemcitabine-resistant PDAC cells and associated with poor outcome of gemcitabine-based therapy [34]. miR-342-3p is regulated by a cross talk between leptin and Notch signaling pathways and increased miR-342-3p resulted in a pro-survival phenotype and an induction of gemcitabine resistance. In contrast, inhibition of miR-342-3p expression increased chemosensitivity to gemcitabine in resistant PDAC cells. Through the utilization of bioinformatics, point mutation analysis, and luciferase reporter assays, researchers identified Krüppel-like factor 6 (KLF6) as a direct target of miR-342-3p. Notably, the introduction of stable KLF6 expression counteracted the impact of miR-342-3p, leading to enhanced apoptosis of pancreatic ductal adenocarcinoma (PDAC) cells when exposed to gemcitabine.

#### miR-296-5p

The oncomir miR-296-5p was shown to be a predictive biomarker for short survival in an analysis performed by Okazaki et al., identifying 2,042 miRNA profiles in cancer tissues from 13 patients with unresectable PDAC [35]. Bioinformatics target analysis with miRDB identified Bcl2-related ovarian killer (BOK), a pro-apoptotic gene, as a target. The transfection of miR-296-5p in various PDAC cell lines led to suppression of BOK, high expression of EMT markers such as vimentin and N-cadherin and decreased apoptosis after treatment with either gemcitabine or 5FU. This suggests a potential role of miR-296-5p in chemoresistance to gemcitabine and 5FU and highlights its association with promoting tumor cell invasion and metastasis of PDAC.

#### miR-17-5p

Similar to other oncomiRs, miR-17-5p was overexpressed in both formalin-fixed paraffin-embedded (FFPE) and microdissected samples of PDAC and associated with poor prognosis [36]. The cell cycle was altered by miR-17-5p via its downstream gene retinoblastoma-like protein2 (RBL2) thereby interacting with the transcription factor E2F [37]. In both in vitro and in vivo settings, the upregulation of miR-17-5p or knockdown of RBL2 disrupted normal cell cycle patterns, leading to accelerated tumor progression and subsequent chemoresistance. Conversely, inhibiting miR-17-5p or increasing RBL2 expression counteracted these effects. The underlying mechanism can be attributed to RBL2’s binding to the promoter regions of consensus E2F target genes. Reduction of RBL2 by miR-17-5p resulted in a shift in E2F activity from gene repression to gene activation, altering E2F’s function from transcriptional balance to proliferation. However, it is plausible that other repressing mechanisms may also be involved. Notably, transfecting PDAC cells with an miR-17-5p inhibitor induced spontaneous apoptosis, increased caspase-3 activation, and heightened chemosensitivity to gemcitabine [38]. Furthermore, inhibition of miR-17-5p upregulated the protein expression of Bim, a pro-apoptotic gene, in a dose dependent manner. However, the mRNA levels of Bim were not changed upon this inhibition, suggesting that miR-17-5p negatively regulates Bim at the post-transcriptional level.

Interestingly, Chatterjee et al. [39] reported contrasting findings regarding the function of miR-17-5p. They observed that miR-17-5p was among the most significantly downregulated miRNAs in paclitaxel-resistant lung cancer cells compared to their paclitaxel-sensitive counterparts. Intriguingly, overexpressing miR-17-5p sensitized the resistant cells to paclitaxel-induced apoptosis and decreased the expression of the beclin-1 gene, which plays a crucial role in modulating autophagy. These conflicting results suggest that the function and prognostic relevance of miR-17-5p may be specific to the tumor type, such as promoting metastasis, as well as treatment related.

#### miR-181-5p

Two independent studies evaluated the predictive value of both serum and plasma miRNA expression for early tumor progression during FOLFIRINOX chemotherapy and its value for stratifying and monitoring PDAC patients [40, 67]. Meijer et al. [40] used micro-array miRNA profiling of plasma samples obtained from patients before and after treatment with FOLFIRINOX. In non-progressive patients, a significant downregulation of miR-181-5p was found which correlated with improved PFS and OS. Moreover, the combination of a decreased miR-181-5p and of the PDAC recurrence marker CA19-9, led to a better correlation with improved survival. However, this combination did not correlate with survival of patients treated with gemcitabine and nab-paclitaxel. miR-181-5p possibly exerts this effect by modulation of the repair of double-strand breaks since it activated ATM, a protein activated during the DNA-damage response (DDR). In vitro inhibition of miR-181-5p enhanced sensitivity to oxaliplatin treatment, likely due to the inhibition of platinum-DNA adduct repair in the nucleus, leading to cell-cycle arrest and enhanced apoptosis. Of note Van der Sijde et al. [67] showed that several serum miRNAs, including miR-194-5p, were downregulated after one cycle of FOLFIRINOX treatment. However, there was no significant correlation between serum miR-194-5p levels and OS, even after adjusting for disease stage, baseline CA19-9 levels, and chemotherapy response. Therefore, miR-181-5p could be considered a more effective prognostic biomarker for assessing metastatic behavior and tumor progression in PDAC patients treated with FOLFIRINOX.

### TsmiRs in PDAC

In contrast to oncomiRs, tumor suppressor miRNAs (Ts-miRs) significantly contribute to the prevention of the tumorigenesis by inhibition of oncogenes in PDAC cells. TsmiRs were found to be downregulated and their enforced expression in PDAC restored their tumor suppressive function. Six potentially clinically relevant and/or commonly studied TsmiRs are discussed in the following section and in Table 1.

#### miR-211

Using unsupervised hierarchical analysis of the data of over 1200 miRNAs in FFPE samples from 19 PDAC patients, miR-211 was identified as the most significant differentiating factor between patients with long OS and those with short OS, with significantly higher expression observed in the long OS group [41]. These findings were further confirmed in microdissected PDAC samples from 60 patients who underwent homogeneous gemcitabine treatment, yielding similar results. In vitro studies in both human PDAC cell lines (AsPc-1, Capan-1, CFPAC-1, HPAC, HPAF-II, MIA PaCa-2, PANC-1, PL45, and Su86.86) and five primary cell cultures demonstrated that overexpression of miR-211 was associated with the antiproliferative effects of gemcitabine, while suppressing miR-211 reduced gemcitabine sensitivity. Moreover, Maftouh et al. demonstrated that high miR-211 expression was characteristic of PDAC cell lines with low metastatic potential, whereas low miR-211 levels were correlated with more metastatic cell lines [42]. Overexpression of miR-211 resulted in decreased expression of its target gene, ribonucleotide reductase subunit 2 (RRM2), which is associated with increased gemcitabine activity and sensitivity in PDAC cells. These findings suggest that miR-211 plays a role in modulating chemosensitivity to gemcitabine in PDAC by targeting RRM2.

#### miR-34a

According to Akula et al. [43], the expression of miR-34a in PDAC specimens was significantly decreased compared to normal tissue. Restoring miR-34a function in p53-deficient PDAC cells led to downregulation of Bcl-2 and Notch1/2 [44]. This resulted in 87% reduction of the tumor-initiating cell population, accompanied by significantly increased growth inhibition by gemcitabine, docetaxel, and cisplatin, as well as increased sensitivity to radiation both in vitro and in vivo indicating that miR-34a plays a crucial role in pancreatic cancer stem cell renewal and cell fate. In addition, in a small study consisting of 24 PDAC patients and 10 healthy controls, serum and salivary miR-34a levels were assessed and it was concluded that, unlike salivary samples which showed no differential expression, serum levels of miR-34 can serve as a non-invasive biomarker for diagnostic purposes [45].

#### miR-146a-5p and miR-30a-5p

A miRNA microarray analysis revealed that miR-146a-5p was significantly decreased in PDAC and correlated with prognosis of PDAC patients [46]. Tumor necrosis factor receptor-associated factor 6 (TRAF6) was validated as a direct target of miR-146a-5p. miR-146a-5p downregulates TRAF6 leading to suppressed PDAC cell proliferation and increased PDAC sensitivity to gemcitabine. Downregulation of TRAF6 by miR-146a-5p also led to downregulation of the whole TRAF6/NF-kB p65/P-glycoprotein axis. Of note, P-glycoprotein works as an efflux pump for many drugs but not for gemcitabine [47, 68], which means that increased sensitivity to gemcitabine should be related to TRAF6 itself. Similar effects on PDAC sensitivity were observed upon upregulating miR-30-5p in PDAC [47]. However, miR-30a-5p was involved in the regulation of another crucial signaling axis, i.e. the FOXD1/ERK axis, which plays an important role in the development of chemoresistance to gemcitabine in PDAC.

#### miR-125a-3p

The function of miR-125a-3p was explored by Liu et al. [48] in the PDAC cells PATU8988T and PANC-1, where they observed a gradual decrease in miR-125a-3p levels upon gemcitabine treatment. In contrast, overexpression of miR-125a-3p suppressed the epithelial-mesenchymal transition (EMT) behavior of PDAC cells and enhanced their sensitivity to gemcitabine. The underlying mechanism involves miR-125a-3p targeting Fyn, a member of the protein tyrosine kinase oncogene family known to be involved in cell growth control and EMT. Notably, the in vitro overexpression of Fyn partially reversed the antitumor effects of miR-125a-3p on gemcitabine chemosensitivity. Collectively, these findings indicate that miR-125a-3p promotes PDAC metastasis through its regulation of Fyn, highlighting also its potential as a therapeutic target to improve gemcitabine response [69].

#### miR-216b

The molecular mechanism underlying the effects of the tumor suppressor miR-216b in PDAC were examined by Liu et al. [49] Consistent with many other TsmiRs, miR-216b was downregulated in PDAC tissues and cell lines. In vitro overexpression of miR-216b inhibited proliferation, migratory and invasive capabilities of PDAC cells. Further analysis identified Rho-associated coiled-coil containing protein kinase 1 (ROCK1) as a direct target gene of miR-216b. Notably, downregulation of ROCK1 suppressed the metastatic capabilities of PDAC cells, which was comparable to the effects observed upon miR-216b overexpression.

## Key roles on miRNAs in PDAC metastatic and chemoresistant phenotypes

**Fig. 2:**
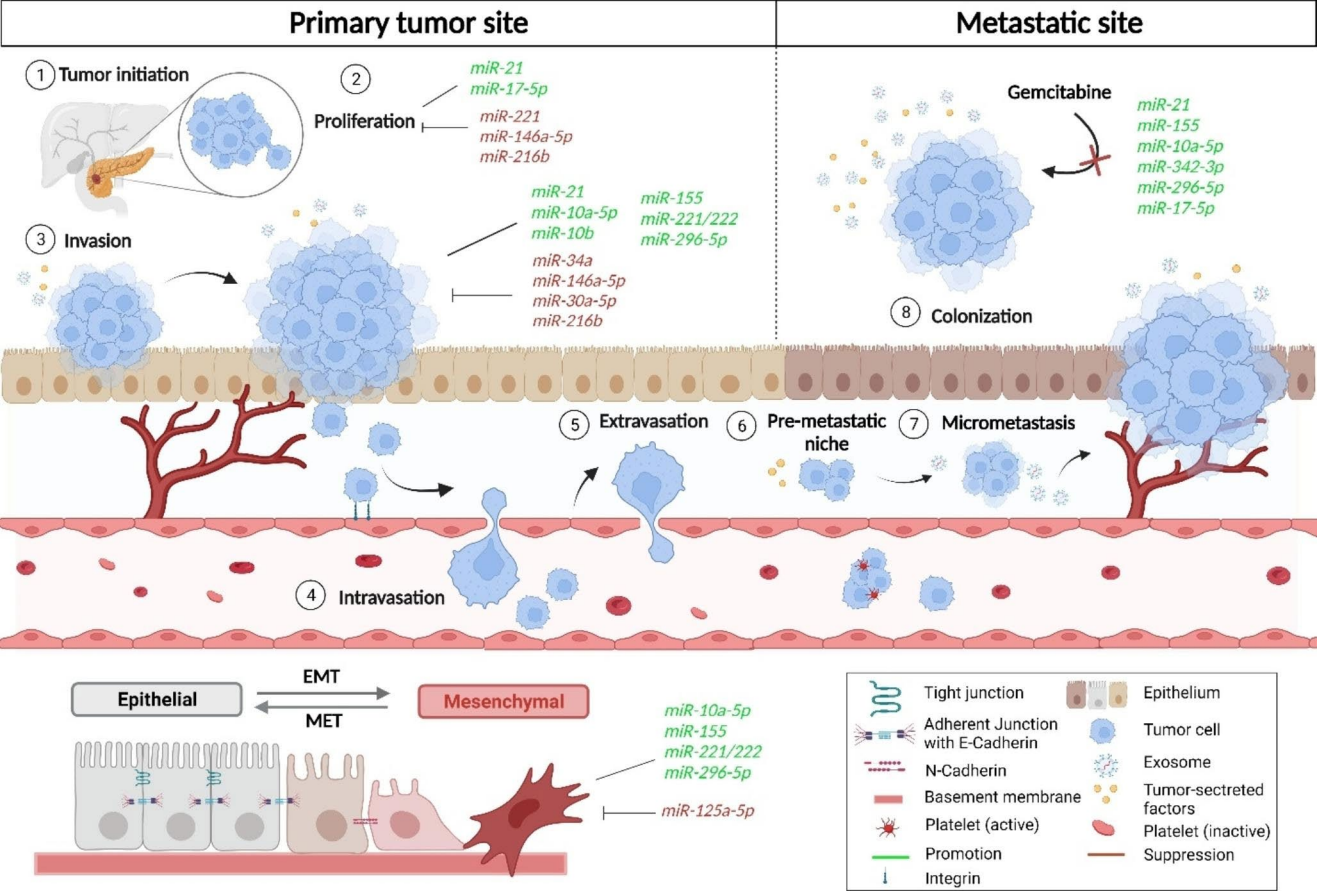
Effect of various oncomiRs and TsmiRs on the various steps of metastasis in PDAC. Different oncomiRs and TsmiRs are involved in the different stages of the tumor progression and metastasis. Numbers (1–8) shows different stages of the tumor progression and metastasis. OncomiRs and TsmiRs are involved in the proliferation, invasion and EMT process. In these stages the expression of oncomiRs increases while the expression of TsmiRs decreases as shown in green and red

Metastatic and chemoresistant phenotypes are distinct characteristics of PDAC, and multiple miRNAs play pivotal roles in the development of such aggressive features. These small non-coding RNA molecules are indeed involved in intricate regulatory networks that modulate key cellular processes contributing to several steps of tumor progression. In particular, miRNAs can influence metastasis by regulating genes involved in EMT, extracellular matrix remodeling, invasion, and migration by altering expression of hundreds of mRNA transcripts patterns in PDAC. A summary of the most important miRNA specifically affecting the various steps of metastasis in PDAC is shown in Fig. 2.

Additionally, several key miRNAs can impact chemoresistance by modulating drug efflux pumps, DNA repair mechanisms, and apoptosis pathways, as previously reviewed by Garajova and collaborators [24]. One example of gemcitabine resistance mediated by miRNA modulation of apoptosis pathway in PDAC cells is highlighted in the study conducted by Giovannetti et al.[50]. Transfecting PDAC primary cell cultures with pre-miR-21, the precursor form of miR-21, was found to have a significant impact on the antiproliferative effects and apoptosis induction by gemcitabine, through modulation of the phospho-Akt pathway in primary cell cultures obtained from resected PDAC patients. Specifically, it led to a decrease in these effects, while when inhibitors targeting phosphoinositide 3-kinase (Pi3K) and mammalian target of rapamycin (mTOR) were added, the phosphorylation of Akt decreased, and the resistance induced by pre-miR-21 to the proapoptotic effects of gemcitabine was prevented.

Lastly, in recent years, there has also been a growing body of research focusing on the role of EVs in the development of PDAC chemoresistance by transporting a diverse range of miRNAs [70]. For instance, pancreatic CAFs release miR-146 and Snail through EVs following exposure to gemcitabine. These EV-packaged Snail and miR-146a are taken up by epithelial cells, promoting the development of chemoresistance. Given these findings, targeting specific EVs could serve as a promising therapeutic approach for patients undergoing gemcitabine-based treatments. Notably, suppressing EVs release from CAFs in vitro reduced Snail expression and impact the survival of resistant cells [71].

Collectively, the pivotal roles of miRNAs in the progression of PDAC and their involvement in resistance to various therapies have prompted extensive research to explore the therapeutic potential of targeting or replenishing miRNAs for PDAC treatment.

## Therapeutic modulation of miRNAs in PDAC

Due to down- or upregulation of miRNAs in PDAC, a promising potential for miRNA-based therapeutics exists. This can be achieved in vivo by modulation of miRNA expression and activity through either miRNA mimics or antimiRs. Multiple innovative therapeutic delivery systems have been developed including viral vectors and nanoparticles such as liposomes that carry miRNAs or antimRs. However, major problems are being encountered including the selection of key miRNAs that are specific for PDAC, and the difficulty to develop efficient and targeted delivery modalities. The various delivery systems should not only transfer anti-miRNAs or miRNA mimics to the tumor, but also enable intratumoral uptake so that they can reach the site of action. Furthermore, some of these carriers such as lentiviral vectors (LVs) might induce toxicity, immunostimulatory effect and off-target effects [72]. On the other hand, anti-miRNAs and miRNA mimics are relatively easy to synthesize as only a limited amount of nucleotides is required to match the mRNA sequence to induce mRNA degradation or translation repression. This benefit comes with a direct drawback of miRNA-based therapy as these strands might bind to wrong mRNAs leading to undesired adverse effects. Additionally, miRNAs that function as tumor suppressors in PDAC e.g. miR-17b have the opposite function in other cancer types i.e. lung cancer which indicates the importance of carefully assessing the role of every miRNA per cancer type. Fortunately, the first studies assessing the potential of miRNA-based therapy in PDAC reported desirable effects of antitumor activity and tolerable toxicities. In the following sections, we describe some of these potentially successful modalities (Table 3) that are now being investigated in preclinical studies to inhibit oncomiRs or to restore the tumor suppressive miRNAs reservoir.

**Table 3:** Overview of delivery systems for miRNAs therapeutics and the effects of their modulation in PDAC

Delivery system	Target	Type study	Effects of modulation of miR	Model	Ref.
PEGylated tandem peptide pTP-iRGD	miR-21	In vitro In vivo	Reduction of PDO size and viability and sensitizing resistant PDOs to gemcitabine Tumor growth suppression of PDX models	Patient-derived-organoid (PDO) of HPDE6c7 epithelial ductal pancreatic cells and PDAC human and mouse cell lines (PANC-1 and D8-175)	[73]
EPOPC:Chol cationic liposomes	miR-21	In vitro	Significant increase in downstream target PTEN, synergistic antitumor effect when combined with sunitinib malate	PDAC cells Hs766T, and normal pancreatic ductal cells HPNE	[74]
EPOPC:Chol cationic liposomes	miR-10b	In vitro	Significant increase in downstream target HoxD10	PDAC cells Hs766T, and normal pancreatic ductal cells HPNE	[74]
EPOPC:Chol cationic liposomes	miR-221 and miR-222	In vitro	Significant increase in downstream target${\text{p}27}^{\text{k}\text{i}\text{p}1}$	PDAC cells Hs766T, and normal pancreatic ductal cells HPNE	[74]
Lentiviral vectors	miR-21	In vivo	Inhibition of proliferation and apoptosis leading to suppressed tumor growth	PDAC Capan-2, MiaPaCa-2 cells and xenograft mouse models	[75]
Nano particles of cationic amphiphile (DOTAP) and co-lipids	miR-34a	In vivo	Enhanced intra-tumoral apoptosis and growth inhibition of subcutaneous cancer xenografts.	MiaPaCa-2 cells and CD-1 athymic nu/nu mice as xenograft models	[76]
Nanoparticles of cationic amphi-phile (DOTAP) and co-lipids	miR-143/145 cluster	In vivo	Enhanced intra-tumoral apoptosis and growth inhibition of subcutaneous cancer xenografts.	MiaPaCa-2 cells and CD-1 athymic nu/nu mice as xenograft models	[76]
Liposomal miR-34a mimic (MXR-34)	miR-34a	Phase I trial	Some antitumoral activity accompanied with tolerated AEs such as fatigue and fever.	Patients aged ≥ 18 years and with refractory solid tumors for which no standard treatment existed	[77]
Exosomes	miR-34a	In vitro	downregulation of miR-34a target gene Bcl-2 and inhibition of pancreatic cells	Normal human pancreatic epithelial ductal cells HPDE6c7, and human PDAC MIA PaCa-2 and Panc28 cell lines	[78]
Exosomes	miR-34a	In vivo	Inhibition of growth of orthotopic PDAC xenografts	Subcutaneous xenograft of Panc28 cells in nude BALB/c mice	[78]

### Inhibiting oncomiRs

Targeting miR-21 has been explored for the past few years, but due to delivery problems to tumor tissue, anti-miR-21 therapeutic use in cancer remained limited. Gilles et al. [73] synthesized tumor-penetrating nanocomplexes (TPN) using nanoparticle carriers coated with oligonucleotide analogs that deliver anti-miR-21 to the tumor site. TPN had been described to increase intratumoral uptake through binding to integrins by tumor penetrating iRGD followed by proteolytic cleavage and initiation of transcytosis through a semaphorin receptor NRP1 which is highly expressed on tumor cells. TPN-21 potently inhibited patient-derived-organoids (PDO) growth of PDAC and even sensitized resistant PDOs to gemcitabine treatment by reducing the organoid size and viability. The use of PDO to predict specific patient response to TPN-21 was also verified in vivo. Patient derived xenografts (PDX) of PDAC cells generated from PDO received repeated intravenous injections of TPN-21. Consistent with the PDO models, tumor growth was suppressed and supported the notion of harnessing such patient avatars to predict clinical outcome. The next step would be a phase I clinical trial for patients with increased miR-21 which would be eligible according to their responsive PDO model. Passadouro et al. [74] designed nano systems by using cationic liposomes coated with human serum albumin to deliver anti-miRNA oligonucleotides targeting overexpressed oncomiRs miR-21, miR-10b, miR-221, and miR-222 to PDAC cells. Silencing of these microRNAs resulted in a significant increase in the levels of their targets i.e. PTEN, HoxD10 and ${\text{p}27}^{\text{k}\text{i}\text{p}1}$ (common target for both miR-221 and miR-222). Interestingly, combination of anti-miR-21 oligonucleotides and low dosage of the chemotherapeutic drug sunitinib resulted in a synergistic antitumor effect represented by a cell viability decrease of 45%. Another in vivo approach to target miR-21 was described by Sicard et al. [75] who administered lentiviral vectors (LV) with RNA interference hairpins antisense to miR-21 by intratumoral injection which resulted in inhibition of miR-21 expression in PDAC cells and tumor necrosis. Despite these promising results, the use of LV might lead to viral immunogenicity, random insertional mutations and activation of oncogenic drivers. Therefore, nano systems carrying miRNAs hold more potential on reaching the clinic as they proved efficient but also safe compared to LV-based therapies.

### Replenishing TsmiRs

Pramanik et al. [76] synthesized lipid-based nanoparticles for systemic delivery of miRNA expression vectors to PDAC cells (nano vectors). Two miRNAs, i.e. miR-34a and a miR-143/miR-145, cluster are downregulated in PDAC and were selected for this nano vector delivery approach. As discussed earlier, miR-34a is functionally involved in the p53 transcriptional network and its overexpression led to downregulation of Bcl-2 and Notch1/2 [44]. The miR-143/145 cluster has an important function in repressing KRAS2 and its downstream effector Ras-responsive element binding protein-1 (RRE B1) [76]. Tail vein injection of both miR-34a and miR-143/145 cluster nano vectors suppressed the growth of PDAC subcutaneous xenografts models. In an orthotopic (intrapancreatic) milieu, the effect was more pronounced with increased cell death and reduced proliferation. Although the expression of the targets Bcl-2 and Notch1/2 was not assessed during this study, nano delivery of miR-34a resulted in significant upregulation of this miRNA as well as downregulation of other specific miR-34a targets such as SIRT1, CD44 and aldehyde dehydrogenase which confirmed the efficacy of this delivery system. Upon intravenous injection of miR-143/miR145 mimics, both KRAS2 and RREB1 were downregulated and no histopathologic or biochemical adverse events were reported supporting testing such nano delivery systems in clinical trials. Indeed, Hong et al. [77] explored the use of a liposomal mir-34a mimic called MRX34 in 47 patients with refractory advanced solid tumors including 5 patients with PDAC. MRX34 was intravenously administered every day for 5 days in 3-week cycles. Treatment with MXR34 was considered relatively effective since some antitumor activity was observed in these patients and the dose-dependent modulation of relevant target genes provided a proof-of-concept for miRNA-based cancer therapy. Most common adverse events (AEs) included fever, fatigue, back pain and nausea. However, the trial was prematurely terminated due to serious immune-mediated AEs that led to the unfortunate deaths of four patients, thereby halting further clinical evaluation of MRX34.

Another successful technique of delivering miR-34a mimics was developed by Zuo et al. [78] who isolated exosomes from HEK293 cells, and used an ultrasound approach to synthesize exosomes-coated miR-34a called exomiR-34a. This exomiR-34a was able to cross the cell membrane efficiently and cause downregulation of miR-34a target gene Bcl-2. Consequently, the growth of the pancreatic cells was inhibited significantly. Furthermore, in vivo xenograft nude mice models bearing Panc28 showed decreased tumor growth. These results shed light on the potential of using ExomiR-34a as a novel anticancer agent for PDAC.

## Conclusions and future prospective

Aberrant expression of miRNAs i.e. upregulated oncomiRs and downregulated TsmiRs play a crucial role in the development, progression and metastases of cancer including PDAC. Moreover, several malignancies have been associated with mutations in the miRNA biogenesis and processing machinery. These mutations can lead to global miRNA deregulation which may promote hallmarks of cancer. Interestingly, recent evidence proposes that oncogenic KRAS mutations modulate the activity of members of the miRNA regulatory pathway and consequently enhance tumorigenesis [79]. miRNAs are now being developed as diagnostic and as predictive or prognostic tools. Moreover, miRNAs have a great potential as therapeutic targets. Despite the considerable and extensive research conducted in preclinical and clinical settings, the integration of miRNA-based applications into clinical practice remains limited, as reviewed by Sempere and collaborators [80]. However, identification of PDAC-specific miRNAs, using also new approaches such as analysis of specific EVs in liquid biopsies, is the starting point towards developing successful treatment strategies.

Unfortunately, naked miRNAs cannot be delivered directly due to their negative charge, short half-life and undesirable off-target and on-target effects. Development of novel innovative delivery systems is in full swing and provide new ideas and directions to treat PDAC. For the development of effective and safe delivery systems, many issues should be addressed before translating miRNA-based therapeutics into the clinic: cellular toxicity, immunogenicity and uptake by tumor cells. For instance, the specificity of nanoparticles delivered to PDAC cells was improved by various strategies such as coupling nanoparticles with specific covalent and non-covalent ligands. These ligands include antibodies, folic acid, transferrin and iRGD peptides that were used by Gilles et al. [73] to facilitate the uptake of the delivery system via overexpressed NRP1 receptors on membranes of PDAC cells. However, developing effective delivery systems for miRNAs requires addressing important considerations such as cellular toxicity and immunogenicity to ensure safe and successful translation of miRNA-based therapeutics into clinical applications. Some potential recommendations include: (1) Optimization of delivery system to minimize cellular toxicity and immunogenicity. This can involve modifying the formulation, surface properties, and size of the delivery vehicles to enhance their biocompatibility and reduce potential adverse effects. (2) Development of specific targeted delivery minimizing off-target effects and enhancing therapeutic efficacy, using strategies such as ligand-receptor interactions. (3) Conduct thorough safety assessments of delivery systems, including comprehensive evaluations of cellular toxicity and immunogenicity in relevant disease models. These studies should assess therapeutic outcomes, pharmacokinetics, biodistribution, immune responses, and potential toxicological effects. Including potential long-term effects on normal tissues or organs. By adhering to these recommendations, there is significant potential to design miRNA-based therapeutics that are safe, efficient, easily producible, and target-specific, making them highly promising for clinical applications. Moreover, exploring possible combinations of anti-miRNAs or miRNA mimics in one delivery system as well as combining miRNA-based therapy with conventional and targeted therapies should be considered. Searching for promising combinations that achieve a synergistic antitumor effect with acceptable side effects would indeed open new horizons to a better personalized treatment approach for patients with PDAC.
